# The spatiality and driving forces of population ageing in China

**DOI:** 10.1371/journal.pone.0243559

**Published:** 2021-01-11

**Authors:** Lianxia Wu, Zuyu Huang, Zehan Pan

**Affiliations:** 1 School of Social Development, East China Normal University, Shanghai, PRC; 2 School of Public Administration, Hunan University, Hunan, PRC; 3 School of Social Development and Public Policy, Fudan University, Shanghai, PRC; Institute of Geographic Sciences and Natural Resources Research (IGSNRR), Chinese Academy of Sciences (CAS), CHINA

## Abstract

Studying the spatial characteristics of China’s ageing and its influencing factors is of great practical significance because China has the largest elderly population in the world. Using 2000 and 2010 census data, this study explores the degree, pace, and pattern of population ageing and its driving mechanism using exploratory spatial data analysis and the geographically weighed regression model. Between 2000 and 2010, population ageing increased rapidly countrywide; yet, spatial differences between eastern and western China narrowed. The degree of provincial population ageing and its spatiality were determined by natural population growth, migration, and local economic development. Life expectancy and mortality were the primary long-term factors, and GDP per capita was the prime contributor in the early days of economic development; the migration rate was the dominant influence after 2010. China’s overall spatial differentiation of population ageing shifted from a north–south to an east–west division.

## Introduction

Population ageing has become a major challenge facing most countries in the world as well as a global research focus for scholars [1–3]. The general decline in fertility rates and the prolongation of per capita life expectancy in both developed and developing countries since 1996 have led to an upsurge in the elderly population around the world. Developed countries were the first experience an ageing society, and they are proceeding toward hyper-aged societies. In contrast, developing countries, despite entering the fray later, are progressing toward ageing societies at an accelerated rate [4]. Several population projections also indicate that the ageing process of the world population will accelerate in the next few decades [5].

In China, the elderly population (aged ≥ 65 years) in 1999 was 86.79 million (excluding the territories of Hong Kong, Macao, and Taiwan), accounting for about 7% of the total population, suggesting that China had entered the ageing society fray. Since then, the elderly population has continued to increase, reaching 158.31 million (11.4% of the total population) by the end of 2017. According to Zhai’s [6] estimation, the elderly population in China should reach a peak of approximately 385 million by 2058 before slightly declining to 308 million by 2100, an increase of 31.54% at its peak.

China is beleaguered not only by having the world’s largest elderly population base and by the rapid ageing of its population but also by vast regional and urban–rural differences in the process of population ageing. However, most extant ageing-related studies focus on predicting the future size of elderly populations, calculating pensions, and estimating the effects of population ageing on economic development from the perspective of demography or economics; rare is the study that considers spatial factors in its exploration of population ageing in China.

Beyond China, the geographical distribution of the elderly, as well as changes to this distribution over time, have piqued the interest of geographical gerontologists who believe that the spatial differentiation of population ageing should have considerable practical and theoretical significance [7]. Exploring the spatial distribution and regional differences of population ageing has, thus, become an entry point for geographers to participate in population ageing research. Early studies, for instance, investigated regional disparities in the distribution of the elderly in many regions, including Toledo, Pittsburgh, Philadelphia, and Baltimore in the United States [8–10]. Furthermore, extant studies have explored the temporal variation of regional disparities in population ageing within a single country (including the Czech Republic, Slovakia, the United States, and Scotland) or made cross-regional comparisons (such as comparative studies of urban and rural areas in England, Wales, Poland, the United States, Italy, Japan, and the United Kingdom) [11–15]. Furthermore, some studies compared the rural and urban differences in sociodemographic, health, and psychosocial factors in an aging Chinese population [16].

The focus of population ageing research has been shifting from merely describing its temporal and spatial variations to ascertaining the mechanisms underlying these variations. Sagaza [17] explored the correlation between fertility reduction and population ageing in the Asia-Pacific region from the perspective of demographic transition. Feng, Son, and Zeng [18] established a positive correlation between positive financial status and successful ageing. Furthermore, making use of data from the Chinese Longitudinal Healthy Longevity Survey (CLHLS) and the Korean Longitudinal Study on Aging (KLoSA), researchers have also found that the advancement of public health systems can assist in halting the progression of noncommunicable diseases among the elderly and help achieve successful ageing in China and South Korea. More broadly, Zhang, et al. [19] have pointed out that China faces two challenges: rapid economic growth and, as the world’s most populous country, population ageing on the largest scale. Hence, the relationship between economic development and population ageing is critical for China.

Although the existing studies have some achievements to their credit for revealing the general trend of population ageing in China and discussing its underlying influencing factors, there is a lack of theoretical generalization given to the temporal and spatial variations in its formation mechanism. Moreover, the policy implications are usually confined to inner-region instead of inter-region. What are the characteristics of temporal and spatial changes in China’s population ageing? What factors have linkages to such changes? How do these linkages vary in the different stages of ageing? Answering these questions can help in understanding the geographical evolution of population ageing and providing a theoretical basis for policy making.

**Fig 1:**
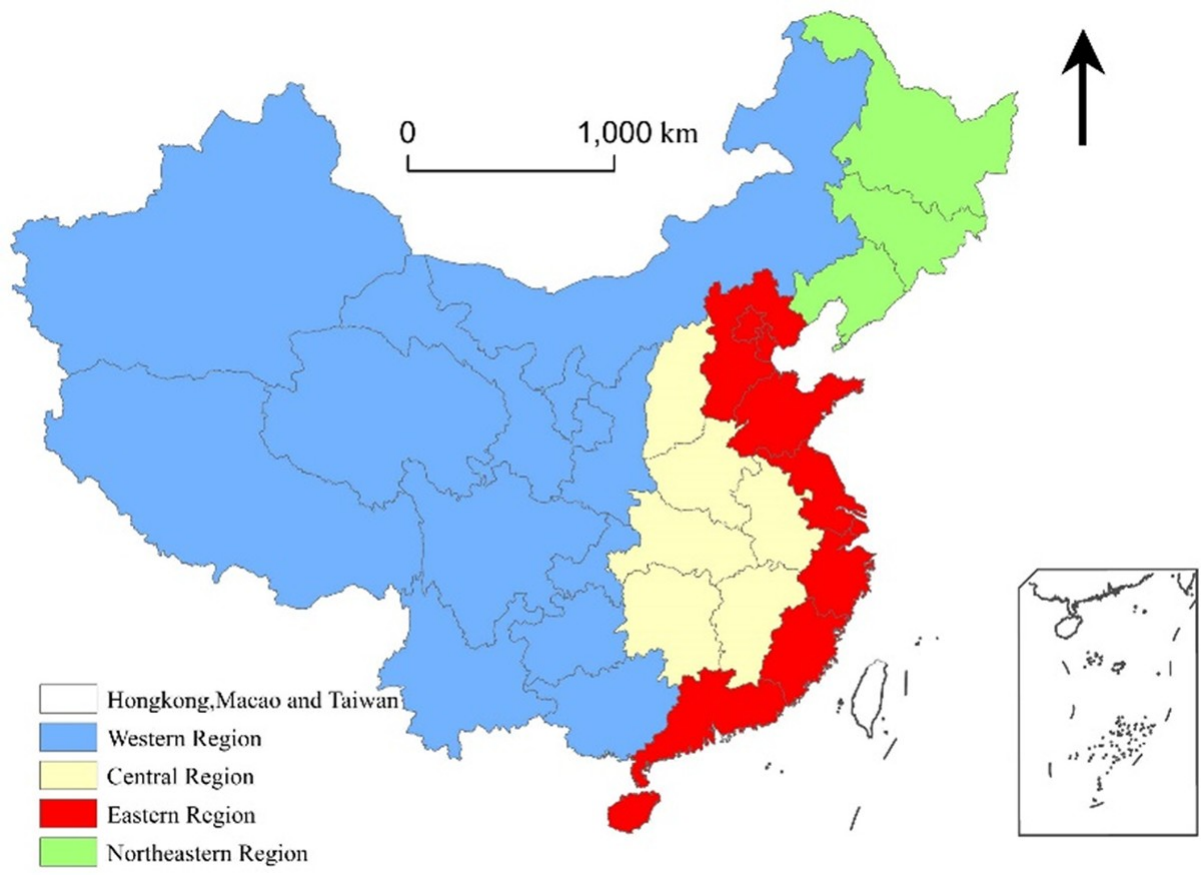
The division of regions in China. Source: This figure was drawn using GIS software.

To answer these questions, our study analyses regional differences in population ageing in three respects—degree, pace, and spatial pattern—and explores the spatial and temporal heterogeneity of the underlying driving mechanisms of population ageing using the geographically weighed regression (GWR) model. Thirty-one provincial administrative regions in China (excluding Hong Kong, Macao, and Taiwan) comprise the basic research unit, and the 2000 and 2010 censuses and the Almanac of China’s Population (2000–2010) constitute the main data sources. For the purpose of making regional comparisons, these provincial administrative regions are divided into four larger areas—eastern, central, western and northeast (Fig 1).

### Regional differences in the population ageing of China

#### Regional differences in the degree of population ageing

The coefficient of ageing, W, is the most commonly used indicator to measure the degree of population ageing; W refers to the proportion of the population aged ≥ 65 years in a certain region at a certain time. Types of population structures can be labelled on the basis of this coefficient: W ≤ 4% signifies that a population is at the youth stage; 4% < W < 7% signifies that the population is at the adult stage; and W ≥ 7% signifies that the population has stepped into the aged stage. To more specifically discuss regional differences in population ageing in China, we further divided the aged stage into three substages according to relevant international standards: early-stage ageing (7% ≤ W ≤ 14%), moderate-stage ageing (14% ≤ W ≤ 21%), and severe-stage ageing (W ≥ 21%). Given below are the results of the cluster analysis of the coefficients of ageing in each province via GIS.

Since the turn of the century, the elderly population has increased more rapidly in most provincial administrative regions, and China, as a whole, has stepped into the early stage of ageing. In 2000, China’s average ageing coefficient was 7.10%, with most provincial administrative regions remaining below the threshold for population ageing and only 14 regions entering the aged stage. In contrast, the average ageing coefficient in China reached 8.92% in 2010, marking an increase of 1.82 percentage points over the decade. At that time, 26 provincial administrative regions entered the aged stage, 11 of which exceeded the country’s average coefficient of ageing. Spatially, the coefficients of ageing in central, western and northeastern regions increased significantly between 2000 and 2010, while the eastern region remained at a constant but high level. Regional differences decreased from 6.99% in 2000 to 6.63% in 2010. Of the 14 provincial administrative regions that entered the aged stage in 2000, only 6 were in central, western and northeastern regions. However, in 2010, with the exception of Tibet, Qinghai, Ningxia, Xinjiang in the west and Guangdong in the east, all other provincial administrative regions had entered the aged stage. In terms of the increase of ageing coefficients, Chongqing recorded the greatest gain (3.71%) among all provincial administrative regions, rocketing from 8.01% in 2000 to 11.72% in 2010. Of the 13 provincial administrative regions with a rate of increase of over 2.0%, except Jiangsu in the eastern region and three provinces in northeastern region, 11 were in the central and western regions. In contrast, of the 5 provincial administrative regions whose rates of ageing were slower, 4 were in the eastern region. Notably, Shanghai was the only region whose ageing coefficient decreased during this period.

#### Regional differences in the pace of population ageing

The old age concentration rate (OACR) and the geographic concentration rate (GCR) can be used to determine the population ageing pace, which is primarily affected by the speed of the transformation of the total population and can be simulated by the exponential growth model. Assuming progressive population ageing complies with the exponential growth model, p(t) (65+) is defined as the population aged ≥ 65 years at year *t* in a certain region. The relevant formulae are as follows:

$$P(t+n)(65+)=e^{n\times r(65+)}\times P(t)(65+)$$ (1)

$$r(65+)=\frac{1}{n}\times \ln\left[\frac{P(t+n)(65+)}{P(t)(65+)}\right]$$ (2)

$$I(t+n)(65+)=e^{n\times TAi(65+)}\times I(t)(65+)$$ (3)

$$SAi(t+n)(65+)=e^{n\times TGi(65+)}\times SAi(t)(65+)$$ (4)

$$TAi(65+)=\frac{1}{n}\times \ln\left[\frac{P_{i}(t+n)(65+)}{P_{i}(t)(65+)}\right]-\frac{1}{n}\times \ln\left[\frac{P_{i}(t+n)(0+)}{P_{i}(t)(0+)}\right]=r_{i}(65+)-r_{i}(0+)$$ (5)

$$TGi(65+)=\frac{1}{n}\times \ln\left[\frac{P_{i}(t+n)(65+)}{P_{i}(t)(65+)}\right]-\frac{1}{n}\times \ln\left[\frac{P(t+n)(65+)}{P(t)(65+)}\right]=r_{i}(65+)-r(65+)$$ (6)

where *P*(*t+n*)(65+) represents the population aged ≥ 65 years in *n* years, r(65+) is the annual growth rate of the population aged ≥ 65 years, *I*(*t*)(65+) represents the proportion of the elderly population in region *i*, while SAi(t)(65+) represents the proportion of the population aged ≥ 65 years in region, making TAi(65+) the OACR. The OACR reflects how rapidly the proportion of the ageing population changes, thereby enabling the determination of differences between changes in the elderly population and changes in the total population of a certain region. The higher the concentration rate is, the faster the ageing process is. Notably, TAi(65+) > 0 shows that the pace of regional ageing is accelerating, TAi(65+) = 0 shows that the pace of regional ageing remains unchanged, and TAi (65+) < 0 shows that the population has not started ageing. The GCR of an elderly population reveals the pace of population ageing in a specific region in relation to the overall national pace. Whether TGi(65+) is greater than, less than, or equal to 0 suggests that the regional pace of population ageing is faster than, slower than, or equal to the national level.

The degree of population ageing, as a static index, can hardly explain the dynamic process of ageing. The computational method created by American demographic statisticians, Andrei Rogers, John F. Watkins, and Jennifer A. Woodward [15] processes and compares the pace of ageing more effectively. In this study, both the OACR and GCR are calculated using formulae (1)–(6); the results are as follows.

**Fig 2:**
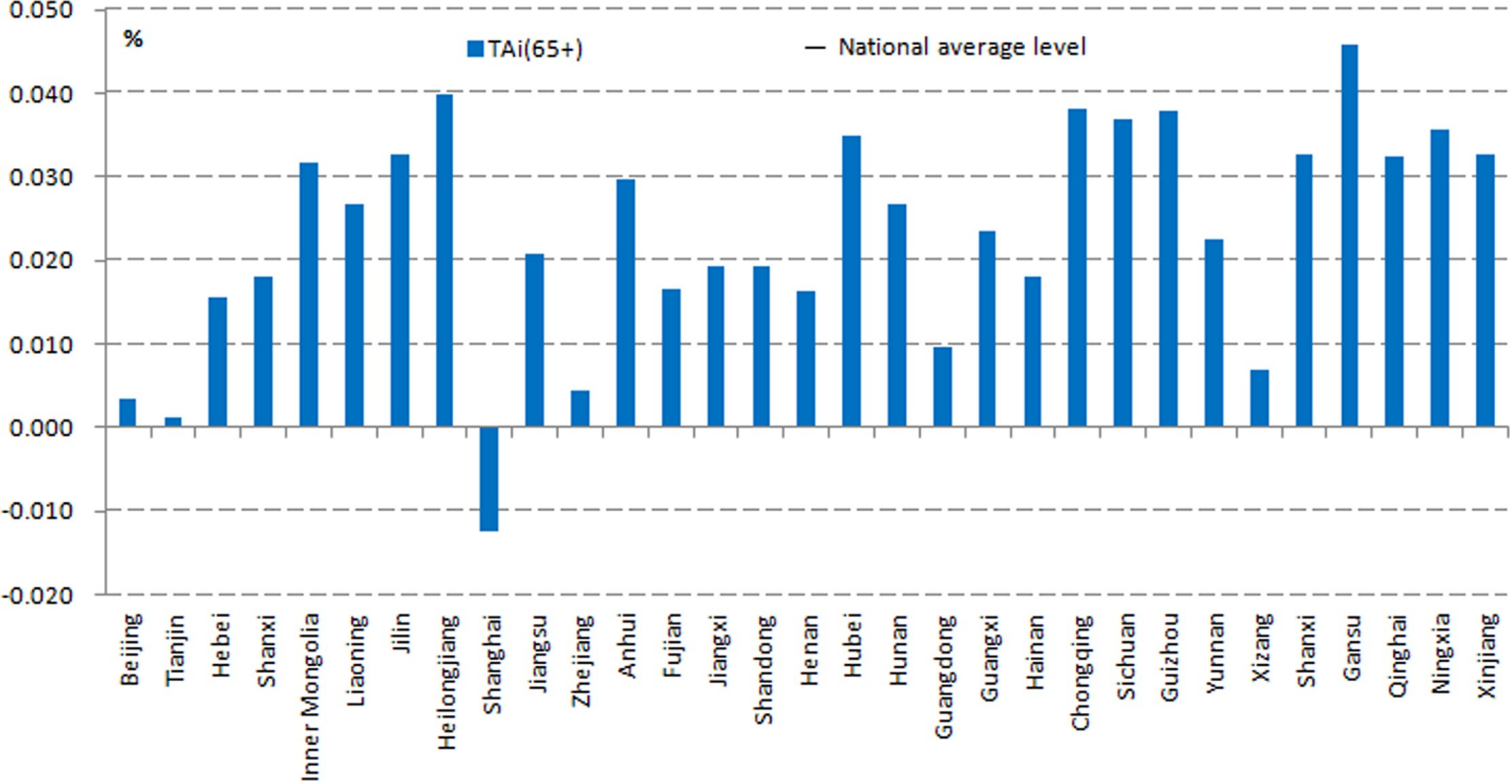
The provincial old age concentration rate during 2000–2010. Source: Census Office of the State Council, (2000). Population census of the People’s Republic of China. Beijing: China Statistics Press; Census Office of the State Council, (2010). Population census of the People’s Republic of China. Beijing: China Statistics Press.

As illustrated in Fig 2, the pace of ageing accelerated in all provincial administrative regions of China between 2000 and 2010. During these 10 years, the OACR in all 30 provincial administrative regions increased (except for in Shanghai), and the growth rates of the ageing population exceeded the growth rate of the total population. In addition, the OACR in 16 provincial administrative regions was above the national level, with Gansu, Heilongjiang, Chongqing, Guizhou, and Sichuan ranked at the top. Note that the OACR in Shanghai was negative, a feasible explanation being the increase in the in-bound migration of young labourers from other areas, which led to the growth rate of its ageing population being marginally lower than the growth rate of its total population.

**Fig 3:**
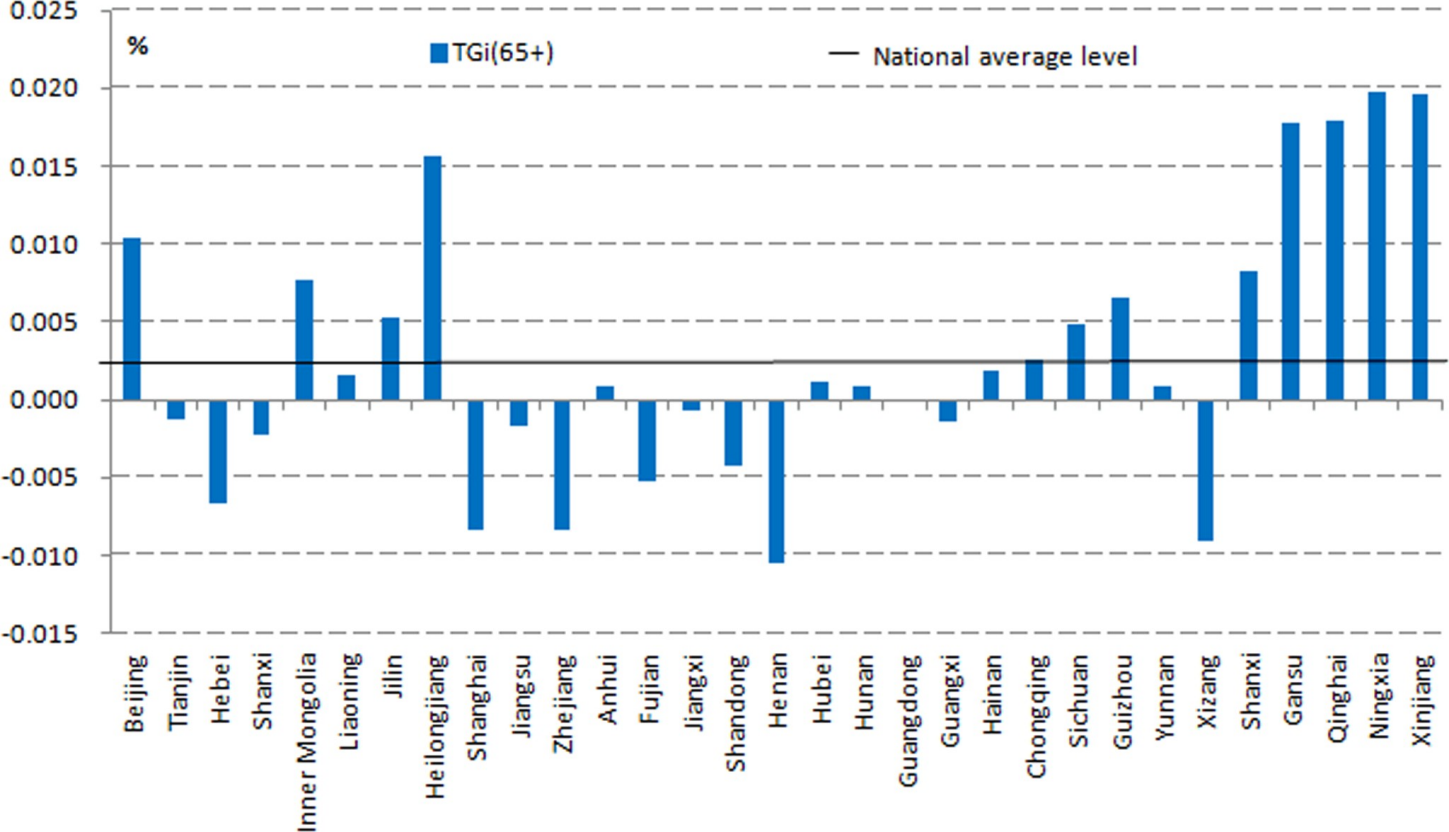
The differences in geographical old age concentration rate during 2000–2010. Source: Census Office of the State Council, (2000). Population census of the People’s Republic of China. Beijing: China Statistics Press; Census Office of the State Council, (2010). Population census of the People’s Republic of China. Beijing: China Statistics Press.

As seen in the GCR (Fig 3), bipolar regional differentiation is evident. Twelve of the regions were below zero, suggesting a slower ageing speed than the national average. The GCR in Henan was 0.0105%—the lowest in the nation—whereas Ningxia was the highest with a GCR of 0.0197%, which was 1.25% higher than the national average.

#### Spatial pattern of population ageing

*Spatial autocorrelation analysis of population ageing*. Exploratory spatial data analysis (ESDA) is a series of spatial data methods and techniques of analysis that have measures of spatial correlation at their core and that describe and visualise spatial distribution patterns. ESDA determines spatial agglomeration and anomalies and reveals spatial interaction between research objects [20].

The visual analysis of the spatial patterns of research objects can uncover anomalies in their spatial agglomeration/dispersion and identify the laws of spatial interaction between them [21]. In addition, global spatial autocorrelation can be used to reveal spatial correlation patterns on a global scale and determine the existence of spatial agglomeration. This correlation is calculated using the following formula [20]:

$$I=\frac{n\sum_{i=1}^{n}(x_{i}-\bar{x})\sum_{j=1}^{n}W_{ij}(x_{j}-\bar{x})}{\sum_{i=1}^{n}\sum_{j=1}^{n}W_{ij}\sum_{i=1}^{n}{\left(x_{i}-\bar{x}\right)}^{2}}\;(i\neq j)$$ (7)

where *n* is the number of observations; *x*_*i*_ and *x*_*j*_ are the observation values of the region *i* and *j*, respectively; $\bar{x}$ is the average value of all regions; and *W*_*ij*_ represents spatial weight. When two regions are adjacent, *W*_*ij*_ = 1; otherwise, *W*_*ij*_ is equal to 0.

Using GeoDa and formula (7) and selecting the proportion of the elderly population as the measurement of ageing, we estimated the global Moran’s I index and spatial correlation index of 31 provincial administrative regions of China from 2000 to 2010.

Table 1 shows that the Moran’s I index values of the proportion of the elderly population both in 2000 and 2010 were above 0, and the normal statistics Z (I) were both higher than the critical value of a 95% confidence level (.96). Thus, both pass the significance test, suggesting that China’s provincial ageing is spatially positively autocorrelated. The Moran’s I value decreased from 0.2406 in 2000 to 0.2139 in 2010, suggesting that the spatial agglomeration of ageing in China has declined, and its spatial correlation has weakened marginally.

**Table 1:** Moran’s I estimation of population aging in China.

Year	Moran’s I	E(I)	sd	Z(I)	P
2000	0.2406	-0.0303	0.1097	2.2489	0.0100
2010	0.2139	-0.0303	0.1161	2.1965	0.0200

Source: This table was created using GeoDa software. The data is from 2000 Population Census of the People’s Republic of China, China Statistics Press; 2010 Population Census of the People’s Republic of China, China Statistics Press.

*Cold and hot spot analysis of population ageing*. Using the Getis-Ord $G_{i}^{*}$ index to differentiate between the high- and low-value clusters in space, the spatial evolution of hot spots is conducive to the comprehensive analysis of the spatial agglomeration of research objects. The Getis-Ord $G_{i}^{*}$ index can be calculated as follows [22]:

$$G_{i}^{*}=\frac{\sum_{j=1}^{n}W_{ij}x_{j}}{\sum_{j=1,j\neq i}^{n}x_{j}}$$ (8)

A standardisation of $G_{i}^{*}$ for better interpretation is the following:

$$Z(G_{i}^{*})=\frac{G_{i}^{*}-E(G_{i}^{*})}{\sqrt{Var(G_{i}^{*})}}$$ (9)

The mathematical expectation and standard deviation of $G_{i}^{*}$ are $E(G_{i}^{*})$ and $Var(G_{i}^{*})$; *W*_*ij*_ is the spatial weight stipulated by the distance rule.

**Fig 4:**
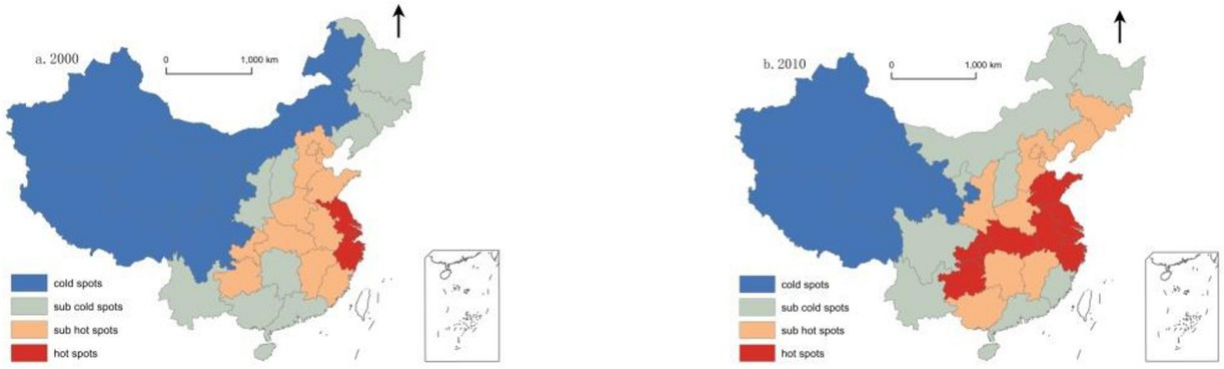
Cold–hot spots analysis on aging (2000–2010). Source: This figure was drawn using GIS software; Census Office of the State Council, (2000). Population census of the People’s Republic of China. Beijing: China Statistics Press; Census Office of the State Council, (2010). Population census of the People’s Republic of China. Beijing: China Statistics Press.

We obtained the local $G_{i}^{*}$ statistics of each provincial region by using the Getis-Ord $G_{i}^{*}$ index and divided every region from high to low into four areas—hot spot, sub-hot, sub-cold, and cold spot—using the natural breakpoint method. Furthermore, we visualised the cold and hot spots of ageing in China using GIS and formula (8), as shown in Fig 4.

In 2000, all ageing hot spots in China were concentrated in Jiangsu, Shanghai, and Zhejiang. In 2010, however, the hot spots were Shandong, Jiangsu, Shanghai, Zhejiang, Anhui, Hubei, Chongqing, and Guizhou. Between 2000 and 2010, the areas with the highest level of ageing and main agglomeration of elderly population in China expanded from the eastern to the central, western and northeastern regions.

In 2000, the ageing cold spots in China were primarily distributed in Xinjiang, Tibet, Qinghai, Gansu, Sichuan, Ningxia, and Inner Mongolia. In 2010, the cold spots were in Xinjiang, Tibet, Qinghai, and Gansu. Between 2000 and 2010, the number of cold spots in China’s ageing was decreasing, and the spatial agglomeration moved from the northwest and northeast to the northwest, suggesting that the degree of ageing in this area was at the lowest level compared to surrounding areas.

## Driving forces of population ageing in China

### The GWR model and indicators

The spatial distribution of population ageing is affected by multiple factors, while the traditional linear regression model (OLS) only makes ‘average’ or ‘overall’ estimates of parameters and fails to illustrate their spatial changes and dependence. As a new model for exploring spatial relationships, the GWR model expands traditional analysis by highlighting the diverse impacts that independent variables have on the dependent variables of different regions. GWR evaluates a local model of the variable or process by fitting a regression equation to every feature in the dataset. It constructs these separate equations by incorporating the dependent and explanatory variables of the features falling within the neighbourhood of each target feature. The shape and extent of each neighbourhood analysed is based on the Neighbourhood Type and Neighbourhood Selection Method parameters. The Neighbourhood Selection Method parameter specifies how the size of the neighbourhood is determined (the actual distance or number of neighbours used). We adopt Akaike Information Criterion (AICc) to select the neighbours [23]. The GWR model is as follows:

$$y_{i}=\beta_{0}(u_{i},v_{i})+\sum_{k}\beta_{k}(u_{i},v_{i})x_{ik}+\varepsilon_{i}$$ (10)

where *y*_*i*_ is the dependent variable of a sample space in China for a year; *u*_*i*_ and *v*_*i*_ are the central coordinates of the number *i* sample of spatial unit; *β*_*k*_ (*u*_*i*_, *v*_*i*_) is the regression coefficient of variable *k* in spatial unit *i*; *k* is the number of variables; *x*_*ik*_ is the value of each variable in a certain year; and *ε_i_* is the error term.

Some studies have proposed that population ageing can be affected by multiple factors [8, 24–26], including population structure, economic living standard, social endowment, social public health, general education, etc. Some studies have identified two critical factors influencing population ageing—the natural growth of a population and migration. The natural growth rate of a population is a representation of the difference between the birth rate and death rate and acts as the inner force affecting population ageing directly, whereas migration is considered an outside force [27]. Besides these two factors, population ageing also has correlations with economic development and level of healthcare [28]. Thus, on the basis of the above references, according to the principles of scientificity, feasibility and availability, this research selected 8 representative factors, including life expectancy, birth rate, mortality rate, in-migration rate, out-migration rate, GDP per capita (represents the economic level, defines the economic level as chivalrous rather than generalized, the same below), the number of beds in medical and health institutions (reflects the level of healthcare) (National Bureau of Statistics of China, 2001–2011), and the proportion of college-educated and above population as independent variables. The percentage of population aged ≥ 65 years acts as the dependent variable.

The coefficient of population ageing in 2000 and 2010 was evaluated by conducting a global spatial autocorrelation analysis; the result reveals that all of the global Moran’s I estimates are above 0, suggesting that the elderly population is aggregated spatially. The local spatial autocorrelation analysis reveals that population ageing exhibits spatial disparities. All these results provide a theoretical basis for the construction of a GWR model to further analyse the impact of different variables on population ageing and the spatial disparities of these variables. The proportion of the elderly population in a certain provincial administrative region in a certain year is assumed to be *y*, and the central coordinate of the number *i* dot is (*u*_*i*_, *v*_*i*_). Hence, on the basis of the above variables being dimensionless, the GWR model can be developed as follows:

$$y_{i}=\beta_{0}(u_{i},v_{i})+\beta_{1}(u_{i},v_{i})x_{1i}+\beta_{2}(u_{i},v_{i})x_{2i}+\beta_{3}(u_{i},v_{i})x_{3i}+\beta_{4}(u_{i},v_{i})x_{4i}+\beta_{5}(u_{i},v_{i})x_{5i}+\beta_{6}(u_{i},v_{i})x_{6i}+\beta_{7}(u_{i},v_{i})x_{7i}+\beta_{8}(u_{i},v_{i})x_{8i}+\varepsilon_{i}$$ (11)

where *β*_0_ represents the intercept, *β*_1_–*β*_8_ represent the estimated regression coefficient of each variable, and *x*_1*i*_–*x*_8*i*_ represent the value of each variable. To compare the differences in the linkages between different independent variables and dependent variable, we nondimensionalize the equations.

### The results of the GWR analysis

In the GWR model, every spatial unit has a specific coefficient. We evaluate the coefficients using formulae (10) and (11). Table 2 presents their average, maximum, and minimum values. As shown in Table 2, GWR illustrates 95.87% of the overall variation in the provincial ageing population in 2000, and 83.61% of the overall variation in 2010. The goodness-of-fit values for the 2000 (0.9337) and 2010 (0.7764) GWR models are a significant improvement over the OLS model (2000 = 0.9154; 2010 = 0.7763). While the OLS analysis provides constant parameters, GWR provides variable parameters that can better represent the spatial variations of the influencing factors so that GWR coefficients illustrate the correlation between ageing and influencing factors more clearly. When the regression coefficient is positive, the two variables are related positively, and when the regression coefficient is negative, they are related negatively. The influence of each factor on the ageing population varies by region.

**Table 2:** GWR parameter estimation and test results.

		Mean value of coefficient	Mean value of coefficient standard deviation
In 2000	life expectancy	0.7438	0.1130
	GDP per capita	0.5045	0.1609
	mortality rate	0.3947	0.0540
	out-migration rate	0.1390	0.0444
	birth rate	0.0581	0.0886
	proportion of college and above population	-0.1025	0.1009
	in-migration rate	-0.0351	0.0892
	number of beds in medical and health institutions	-0.0065	0.0520
In 2010	life expectancy	0.6430	0.1922
	mortality rate	0.5574	0.1185
	in-migration rate	0.3653	0.2079
	out-migration rate	0.2200	0.1077
	number of beds in medical and health institutions	0.0677	0.0980
	GDP per capita	-0.3377	0.2514
	birth rate	-0.2554	0.1082
	proportion of college and above population	-0.0435	0.2101
		In 2000	In 2010
	*R*^2^	0.9587	0.8361
	adj.*R*^2^	0.9337	0.7764

Note: Arranged according to the positive–negative and the size of the mean value of the coefficient in different years (for easy comparison, the four bits after reservations are reserved).

Sources: (1) Census Office of the State Council. (2000). Population census of the People’s Republic of China. Beijing: China Statistics Press; Census Office of the State Council. (2010). Population census of the People’s Republic of China. Beijing: China Statistics Press. (2) National Bureau of Statistics of China. (2001). China statistical yearbook. Beijing: China Statistics Press; National Bureau of Statistics of China. (2011). China statistical yearbook. Beijing: China Statistics Press.

### Influencing factors of regional differences in population ageing

The leading factors that affect the ageing population in China changed over time. The mean value of each factor depicts the overall relationship between that factor and China’s ageing population. The coefficient standard deviation reflects the spatial imbalance of factors in different provincial administrative regions.

#### Year 2000

Life expectancy is the primary factor that affected ageing in 2000. Longer life expectancy correlated with a larger elderly population; the greater the proportion of the elderly population, the more absolute ageing is promoted, accounting for a more serious ageing problem. Absolute ageing is the ageing of the increase of the elderly population and consists of two aspects: the longevity of the elderly population and the ageing of the new population. Demography refers to this as ‘top ageing’ based on the change to population age structure. Longevity refers to an increase in the number of long-lived people, and the ageing of the new population means that the number of people entering old age increased each year. This is the follow-up effect of the past birth peak; it has become the current growth peak in the old age population.

**Fig 5:**
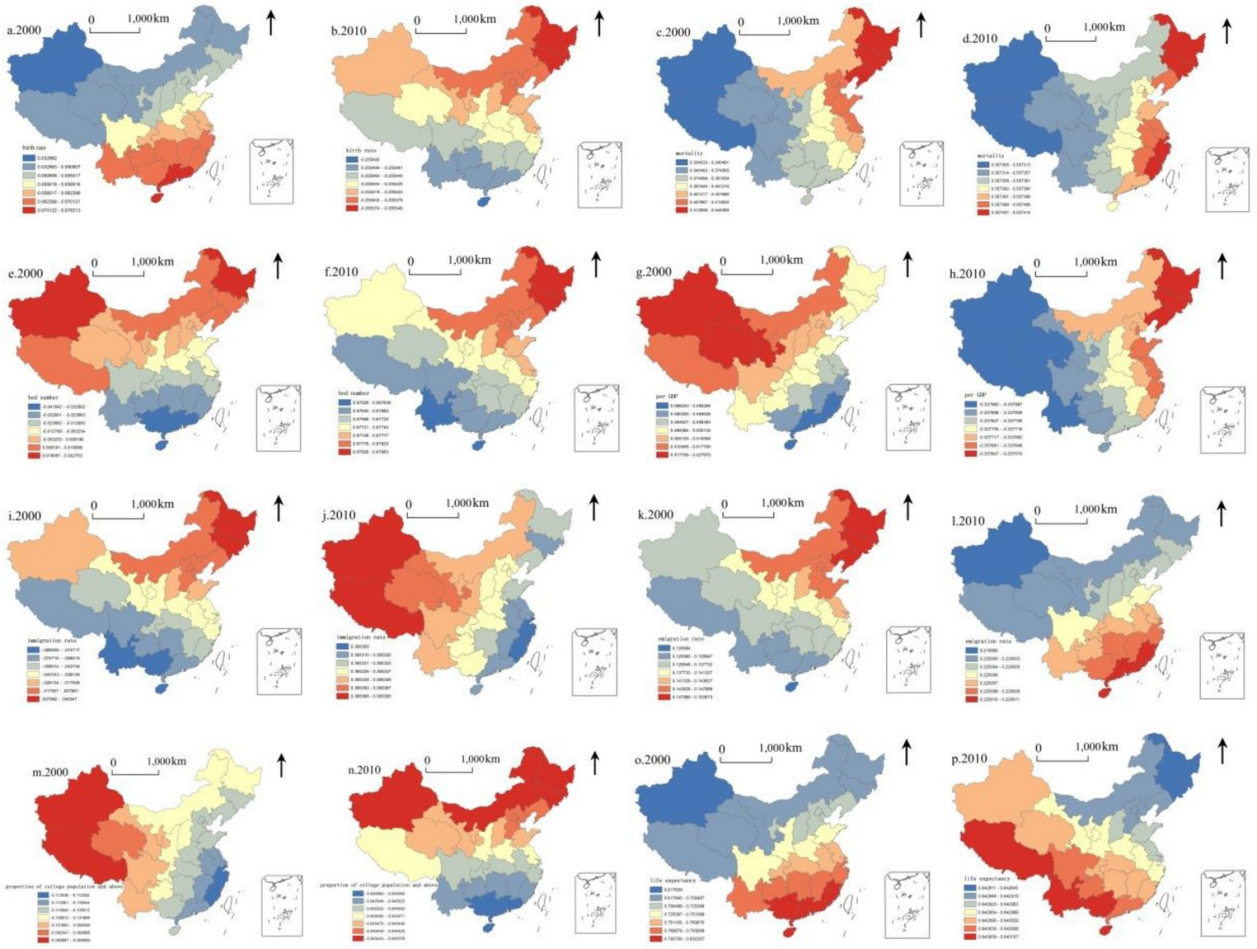
Spatial distribution of regression coefficients based on the GWR model in 2000 and 2010. A, c, e, g, i, k, m, and o = birth rate, mortality rate, number of beds in medical and health institutions, GDP per capita, in-migration rate, out-migration rate, the proportion of college population and above, and life expectancy, respectively, in 2000. B, d, f, h, j, l, n, and p = birth rate, mortality rate, number of beds in medical and health institutions, GDP per capita, in-migration rate, out-migration rate, the proportion of college population and above, and life expectancy, respectively, in 2010. Sources: (1) Census Office of the State Council. (2000). Population census of the People’s Republic of China. Beijing: China Statistics Press; Census Office of the State Council. (2010). Population census of the People’s Republic of China. Beijing: China Statistics Press. (2) National Bureau of Statistics of China. (2001). China statistical yearbook. Beijing: China Statistics Press; National Bureau of Statistics of China. (2011). China statistical yearbook. Beijing: China Statistics Press.

GDP per capita was the second leading factor affecting ageing. The coefficients of all factors are unbalanced, and generally, the Yangtze River is the boundary. Of all the influencing factors, the spatial heterogeneity of the coefficient of GDP per capita is the most apparent, exhibiting spatial differences between north and south, which corroborates differentiation in economic development between the north and south during the same period (Fig 5).

In its early stages, economic development played a decisive role in promoting ageing (the mean value of the coefficient is 0.5045, which is much higher than that of all the other factors except life expectancy). The level of economic development not only augmented the living standard and quality of medical care but also extended life span to a certain extent, promoting absolute ageing. In addition, economic development inhibited relative ageing by enhancing the quality of life of the population and decreasing the desire to give birth. Relative aging refers to an increase in the proportion of elderly due to a decrease in the birth population caused by family planning or out-migration of the young population. The former is called ‘bottom ageing’ in demography, and the latter ‘waist ageing’.

Moreover, regional economic disparities led to migration, which also influenced the spatial layout of the elderly population. Typically, the more developed a regional economy was, the more likely young people were to choose to move to that region (with the young population dominant) and consequently lower the degree of the ageing. Conversely, the less developed an economy was, the more likely the young population was to leave and accelerate the ageing process.

The coefficient of the GDP per capita decreased from north to south. The economy played a decisive role in the ageing process, whereas the level of most other factors remained relatively low, exerting little impact on ageing. This situation is apparent primarily in the northwest region. For example, in Qinghai, the GDP per capita was only 5,087 RMB, fewer people left (94,988), the out-migration rate was only 1.97%, medical care was relatively poor (the number of beds in medical and health institutions totalling only 20,000), life expectancy as only 66.03 years, and the overall quality of life was low. Thus, it backward economic development that primarily determined the lagging population growth stage (a high birth rate, high mortality rate, and high natural growth rate) and prevented the age structure of the population from entering the aged stage (the ageing rate is only 4.50%).

However, when the economy as relatively developed, its impact on ageing was smaller than it was in north-western China. The comprehensive impact of positive and negative effects of large-scale in-migration, the enhancement of medical and educational conditions, and the relatively high rate of natural growth resulted in the differentiation of population ageing patterns. The negative effects were higher than the positive and inhibited the progress of ageing. For instance, in Heilongjiang, the GDP per capita was 8,562 RMB, and although the quality of life and medical care were enhanced (120,000 beds), the negative impact of mass in-migration (386,641 people) and the high natural growth rate (3.36‰) restrained population ageing (the ageing rate is only 5.2%). In addition, the positive effect was higher than the negative, and promoting the development of ageing.

In Shanghai, for instance, the in-migration rate was the highest in the country (19.11%), which inhibited ageing. However, its natural growth rate was the lowest in the entire country (–0.27‰), resulting in a relatively high level of ageing. Meanwhile, Shanghai’s advanced economy (GDP per capita is 34,574 RMB) led to increased quality of life, increased availability of medical and health services (80,000 beds), and prolonged life expectancy (78.14 years), thus, accelerating the ageing process. The blend of positive and negative factors played a vital role in promoting ageing in Shanghai. These comparisons show that the fundamental factors that affected the ageing process and its spatial differentiation in 2000 are economic development and its spatial differences.

#### Year 2010

Both in-migration and out-migration emerged following life expectancy and mortality rate in 2010. In addition, GDP per capita changed to be the principal negative factor. The coefficient of every factor presents spatial imbalance. Of all factors, the standard deviation of the coefficient of GDP per capita and in-migration rate is largest, and thus their spatial heterogeneity is strongest. Notably, the difference in the role of each factor is roughly divided by the central region, exhibiting a gradual east–west change trend, which corroborates the pattern of migration and economic development of China during this period (Fig 5).

Life expectancy and mortality became the leading factors correlated with ageing as the rapid development of social economy since the reforms and opening-up enhanced quality of life and medical care. In 2010, the decline in life expectancy progressed from the southwest to the northeast. Life expectancy was longer in Tibet, Yunnan, Guangxi, and Hainan; this trend was reversed in Heilongjiang and Jilin. The decrease in the mortality rate progressed from the east to the west. Heilongjiang–Jilin and Zhejiang–Fujian were the two areas with the highest mortality rates. The areas with low mortality rates were Xinjiang and Tibet. The higher the mortality rate, the more serious the ageing population situation was, demonstrating that the regions with higher mortality rate were there wither high ageing rate. This result reflected that the reverse effects of ageing rate on mortality rate may be dominant in the cycle casual relationships between them.

Lately, population migration became a crucial correlating with ageing, and its linkage to ageing increased sharply. In 2000–2010, the in-migration proportion in the eastern regions increased to 80.74%. Both central, western and northeastern regions remained the largest out-migration areas in China, with an out-migration proportion as high as 81.52% (Table 3). (1) The out-migration rate primarily promoted the ageing process in the central, western and northeastern regions and its leading role was increasing. The impact of the out-migration rate on the ageing of net out-migration intensified more with the relaxation of population migration controls and the widening of the scale of migration. (2) The in-migration rate decreased the dilution power of ageing in the eastern region, and migration remained active. The dilution ratio of the in-migration rate to ageing in the eastern region decreased and transformed into a promoting force. Population migration remained active. In-migration comprised not only young people who had graduated from junior college and above (early marriage age, low birth intention, low birth rate, and low natural growth rate) but also, progressively, the elderly population (owing to increasing pressures to care for children in China). This decreased the growth rate of ageing but, at the same time, promoted mutual superposition and the common development of relative and absolute ageing. Thus, under the combined effect of the factors of in-migration rate and out-migration rate, the growth rate of ageing in the eastern region was the lowest, but in the central, western and northeastern regions, it was more rapid and serious, even as the old age dependency ratio increased rapidly.

**Table 3:** The interprovincial in-migration and out-migration in four regions of China in 2000 and 2010.

		China	Eastern region	Central region	Western region	Northeastern region
Population number (tens of thousands of people)	trans-provincial in-migration in 2000	4,241.86	3211.45	258.54	597.83	174.04
	trans-provincial out-migration in 2000	4,241.86	719.90	1849.33	1458.16	214.04
	trans-provincial in-migration in 2010	8,587.63	6933.44	415.45	1041.07	197.66
	trans-provincial out-migration in 2010	8,587.63	1586.65	3970.86	2756.94	273.19
proportion (%)	trans-provincial in-migration in 2000	100.00	75.71	6.09	14.09	4.10
	trans-provincial out-migration in 2000	100.00	16.97	43.60	34.38	5.06
	trans-provincial in-migration in 2010	100.00	80.74	4.84	12.12	2.30
	trans-provincial out-migration in 2010	100.00	18.48	46.24	32.10	3.18
in-migration rate or out-migration rate(%)	trans-provincial in-migration in 2000		1.63	0.58	1.79	0.58
	trans-provincial out-migration in 2000		2.01	4.13	4.37	4.13
	trans-provincial in-migration in 2010		1.69	0.87	3.02	0.87
	trans-provincial out-migration in 2010		2.33	8.33	8.01	8.33

Source: 2000 Population Census of the People’s Republic of China, China Statistics Press; 2010 Population Census of the People’s Republic of China, China Statistics Press.

Having been supplanted by absolute ageing, the ageing process accelerated faster than economic growth. In addition, the number of economically insecure elderly (‘not getting rich before getting old’) became more prevalent, and the promotion effect of GDP per capita on absolute ageing further debilitated. In economically developed areas, an upsurge in the cost of living, especially in the cost of childbirth and raising children, led to a corresponding decline in people’s fertility willingness, which aggravated the decline in the birth rate. When ageing increased to a certain extent, it, in turn, exerted several adverse effects on the further development of productive forces and scientific and technological innovation, causing a serious impediment to economic growth and scientific–technological progress. Thus, economic development began to ‘decouple’ from the overall level of ageing or even to become negatively correlated.

When the economy had developed to a certain level, the impact of ageing gradually decreased and transformed from a driving force to a hindrance. When there was ‘elderly economic insecurity ‘ or ‘decoupling’ from ageing, economic development negatively correlated with the level of ageing. The former was observed, for instance, in Chongqing, where the GDP per capita was 27,596 RMB (less than the national average), but the degree of ageing was the highest in the country (11.56%); this is primarily attributable to spatial differences in economic development, which resulted in a net out-migration of > 1.96 million people. The latter was observed, for instance, in Shanghai, where the economic level was high, economic growth was rapid, GDP per capita remained the highest in the country for a considerable period of time (34,547 RMB in 2000 and 76,074 RMB in 2010), quality of life was improving, and fertility willingness declined. This has resulted in low or even negative population growth (–0.27‰ in 2000 and 1.98‰ in 2010), even as the rate of ageing was declining (from 11.46% in 2000 to 10.13% in 2010).

In Shanghai, the correlation between economic level and ageing was insignificant or even negative. However, the more backward the economy was, the slower the economic growth rate and the higher the out-migration rate, leading to a relatively higher ageing population. Meanwhile, the lower the level of economic development was, the lower the quality of the population demographics, the more backward the concept of fertility, and the higher the birth rate. Additionally, the more backward the quality of medical care was, the higher the crude mortality rate of children or the working age group, and the higher the rate of ageing. For instance, in 2010, Guizhou’s GDP per capita was the lowest (13,119 RMB) in the country; the out-migration rate was 11.65%, much higher than the national average of 6.44%; the proportion of college-educated and above population was only 5.29%, the lowest in the country; the birth rate was relatively high at 1.396%; the quality of medical care was regressive (the number of medical beds was only 110,000, lower than the national average of 150,000); the mortality rate was approximately 6.55‰; and the ageing rate was relatively high at approximately 8.71%.

The impact of the birth rate on ageing changed from a weak positive effect to a strong negative effect, and the impact strength began to increase. From 2000 to 2010, the concept of childbearing was improved by raising the education level of females (a 15.47-million increase in undergraduate females) and the implementation of the family planning policy; this, in turn, decreased the birth rate by 2.13% even as ageing increased. From 2000 to 2010, the birth rate in Hainan exhibited the highest impact (the absolute value) on ageing, 0.078313 and 0.255495, respectively, with 2010 showing the highest negative effect. However, the birth rate (14.58‰) and ageing rate (6.74%) in 2000 were, respectively, lower than the birth rate (14.71‰) and ageing rate (8.07%) in 2010. Hainan’s GDP per capita was 6,894 RMB (in 2000) and 23,831 RMB (in 2010), making it one of country’s underdeveloped economic areas. The birth rate in Heilongjiang was 7.35‰, the ageing rate was 8.28% in 2010, and the negative impact of the birth rate on ageing was the highest in the country (–0.255340; the absolute value was the lowest in the country).

In 2000, the birth rate positively correlated with ageing; that is, the higher the birth rate was, the more newborns in the region. However, the impact of the birth rate on ageing was relatively small, and the high proportion of the ageing population in the region can be attributed to several other causes, such as a large outflow of young people and a large inflow of older adults. In 2010, the number of newborns in high-birth-rate areas was considerable, and the proportion of the low-age population was relatively high; thus, the proportion of the elderly population was relatively low. In addition, the proportion of newborn children in the low-birth-rate areas was low, accounting for the increased elderly population. From 2000 to 2010, the impact of the birth rate on ageing transformed from positive to negative, with the positive effect decreasing from the south to the north and the negative effect decreasing from north to south (its absolute value also decreased from south to north), indicating that the more backward economic development was, the higher the impact of the birth rate on ageing. Conversely, the more developed an economic area was, the lower the birth rate, and the impact of the birth rate on ageing for these areas was far lower than for less developed areas. Moreover, the homogenisation effect of the birth rate demonstrates the rigidity of the family planning policy as a basic national policy. Since the implementation of the family planning policy in 1973, the birth rate has decreased rapidly in all provinces. Until 2010, the proportion of children was reduced markedly, and the ageing process accelerated; however, this effect was weaker than absolute ageing during the same period.

Education level weakly negatively correlated with ageing. Typically, relative ageing of the population was promoted by having a higher proportion of people who were college-educated and above, whose first marriages took place later in life, and who, therefore, had lower fertility. However, a higher proportion of people who were college-educated and above (primarily young people and thus reflecting a higher proportion of young people) correlated with lower relative ageing.

The proportion of the young and highly educated population in the earlier period was not significant to regional ageing; however, because of historical inertia, the proportion of the college-educated and above population in the northern region remained high, whereas it remained low in the southern region and negatively correlated with the regional ageing rate, with the highest negative correlation in the southern region. Affected by the population growth queue effect and the period effect, by 2000, the birth population of the three birth peaks (1949–1957, 1962–1970, and 1980–1990) entered the young group in succession. The superposition of the three peaks markedly elevated the absolute population in this age group, resulting in a ‘demographic dividend’ during the peak period. Consequently, the sustained and rapid decline of the birth rate and the proportion of children in the provinces in this period primarily corresponded to the rise in the proportion of young and middle-aged groups, even as the correlation with birth rate and ageing was relatively small, especially in the eastern region, which had a high ageing rate and low birth rate.

The quality of medical care positively correlated with ageing and its impact was rising. In Xinjiang in 2000, the number of beds at medical and health institutions (70,000) and the level of ageing (4.53%) were relatively low, and the mortality rate was relatively high; the region had not yet even entered the aged stage. In 2010, with an increase of 1,610,000 beds in national medical and health institutions, as well as an increase of 15.26 million basic old-age insurance policies, the ageing process accelerated. Thus, higher levels of medical treatment promoted ageing.

Overall, the agglomeration of population migration fundamentally restricted the spatial pattern of ageing, and life expectancy and mortality were further strengthened to promote the development of ageing in 2010.

## Discussion and conclusions

The degree of ageing population in China increased between 2000 and 2010, mostly in the primary stage of ageing, and regional differences in ageing narrowed. Ageing first occurred in the relatively developed eastern regions, especially in such municipalities as Shanghai and Chongqing. Ageing has become more widespread in the central, western and northeastern regions; in the eastern region, it remains high and has begun to divide. The ageing rate of the entire country has increased recently (except for Shanghai), and the difference in the ageing rate across the areas remains significant. The spatial difference in ageing between east and west consistently declined, the spatial agglomeration effect decreased, high-value clusters of ageing increased, and low-value clusters decreased; meanwhile, high-value clusters expanded from the eastern region to the central, western and northeastern regions, but low-value clusters from the northwest and northeast concentrated in the northwest.

Overall, the ageing process of the provincial populations and its spatial differentiation result from the comprehensive impact of three factors: death, birth, and migration. The absolute ageing of death-related factors was most dominant and birth-related factors secondary, with migration-related factors playing a crucial moderating effect. In different periods, the specific leading factors and their mechanisms of action differ, which has resulted in the evolution of regional imbalances in China’s ageing.

As shown in Table 4, both life expectancy and mortality rate are the leading factors for the long-term development of ageing. The GDP per capita in the early period of economic development (in 2000) played a major role in ageing. What is more, it may have resulted from economic development during the 1990s, which had a temporal lag effect. With continuous economic development and large-scale migration, the in-migration rate began playing a major role in ageing in 2010.

**Table 4:** The main types of mechanisms and the influence ranking for the period.

Mechanism types		Period (Influence ranking)	
		In 2000	In 2010
**Death**	Life expectancy(+)	1	1
	Mortality rate(+)	3	2
	GDP per capita change from(+)to(–)	2	4
	Number of beds in medical and health institutions Change from(–)to(+)	8	7
**Birth**	Birth rate change from(+)to(–)	6	5
	Proportion of college and above population(–)	5	8
**Migration**	In-migration rate change from(–)to(+)	7	3
	Out-migration rate(+)	4	6
	Proportion of college and above population(–)	5	8

Economic development is one of the main driving forces of the increase in life expectancy, the decline in fertility and mortality rates and the changes in migration rate. In other words, economic development may influence ageing rate through these factors. Thus, the effects of economic development may have a lag in time behind the direct effects of fertility, mortality, life expectancy and migration. Hence, the correlation between GDP per capita and ageing rate reflects the earlier influences than the four factors. Before 2000, economic development markedly promoted the proportion of the elderly population in the early-development regions through improving life expectancy, suppressing fertility rate and mortality rate. Between 2000–2010, the regional disparity of economic development induced large-scale labourer migrants towards the regions with higher income and more job opportunities. The internal migration suppressed the ageing rate in the destination regions and increased the ageing rate in the original regions. Hence, the effects of GDP per capita changed to be negative. Finally, as the urbanization rate rose and the scale of internal migrants fell, the economic disparity shrank and the reduction effects of migration weakened. The regions with higher ageing rate might have more migrants stock and higher mortality rate. The instant linkages between life expectancy, fertility and mortality with ageing rate presented tighter.

The change in the birth rate has significant human control characteristics, which exert a rigid and homogeneous impact on the ageing population in China. By restraining the proportion of the population in the children’s group, the relative ageing was accelerated; this was especially true when the population growth of China’s young and middle-aged groups exceeded the peak value and the low birth rate accelerated the ageing of the provincial population. Nevertheless, the rapid development of social economy is the fundamental reason for ageing. Owing to the improvement of life and medical conditions, even if family planning had not been implemented, with socioeconomic development, China would still have entered the aged stage.

The increasingly significant regional agglomeration of migration has resulted in the formation of a spatial pattern of regional differences in ageing; the primary mechanism in this pattern is that the population age structure of interprovincial migration is the young. The active migration of the labour population is conducive to the inhibition of the ageing process of economically developed areas in the east and southeast and the accelerating development of ageing in central, western and northeastern regions, whereas the problem of ageing in central, western and northeastern regions is increasingly severe, and the dependency ratio of the elderly population is increasing rapidly. At the provincial level, the effects of the in-migration and out-migration rates on ageing have been strengthened, resulting in the adjustment role of migration in the ageing of China’s provincial regions, which was further strengthened in 2010.

Based on this study, we wish to propose some policy changes for coping with the trend of ageing: first, it is suggested to break down the barriers of regional policies and develop the inter-regional cooperation, like the integrated development strategy of pension services in the Yangtze River Delta; second, strengthen the guidance of population migration and adjust the number and direction of inter-provincial migration; third, implement differentiated family planning policy on the basis of the characteristics of population aging in different regions, such as relaxing "comprehensive two-child" policies in the regions with high levels of ageing; fourth, develop flexible retirement system and support the development of the elderly industry; fifth, promote the "healthy China" strategy, including encouraging not only elders but also youngsters to take part in physical exercise, develop good living habits, maintain a healthy lifestyle and spiritual quality of life.

## Supporting information

S1 Data (XLSX)
